# Persistent, Poorly Responsive Immune Thrombocytopenia Secondary to Asymptomatic COVID-19 Infection in a Child

**DOI:** 10.1155/2023/3298520

**Published:** 2023-12-15

**Authors:** Chamila Mettananda, Senani Williams

**Affiliations:** ^1^Department of Pharmacology, Faculty of Medicine, University of Kelaniya, Colombo, Sri Lanka; ^2^Department of Pathology, Faculty of Medicine, University of Kelaniya, Colombo, Sri Lanka

## Abstract

Immune thrombocytopenic purpura (ITP) secondary to asymptomatic COVID-19 infection, especially in children, is not reported. Furthermore, persistent, treatment-resistant ITP secondary to COVID-19 is not reported. We report a previously healthy 14-year-old Asian boy who developed secondary ITP following an asymptomatic COVID-19 infection and is having a relapsing and remitting cause with poor response to immunosuppressants even after 21 months following the diagnosis. This case emphasizes the importance of testing for COVID-19 in newly diagnosed ITP patients and the need for follow-up platelet counts in patients who recover from COVID-19 as it may follow into developing secondary ITP yet being asymptomatic until you present with a bleeding complication of ITP. The poor response to standard immunosuppression warrants more understanding of the pathophysiology of persistently low platelets following COVID-19 infection. Long-term sequelae of the disease highlight the importance of getting vaccinated for COVID-19 despite COVID-19 being no longer a global emergency.

## 1. Introduction

Thrombocytopaenia is commonly observed in acute COVID-19, but immune thrombocytopenic purpura (ITP) following COVID-19 is not common. ITP secondary to COVID-19 is seen more in males and the elderly. Most cases developed ITP within 2-3 weeks after the COVID-19 infection and recover in less than one week from ITP [1, 2]. It is less common among children and is not reported following an asymptomatic COVID-19 infection in children. Furthermore, persistent ITP beyond a few months is not reported.

We report a previously healthy teenage Asian boy who developed ITP following an asymptomatic COVID-19 infection and responded partially to the standard immunosuppressive treatment was having low platelets even at 21 months from the onset.

## 2. Case Presentation

A previously healthy 14-year-old Asian boy presented with a one-day history of fever and a platelet count of 18 × 10^9^/L in September 2021 during a local COVID-19 outbreak. His full blood count done 18 months ago when he presented with a fever was completely normal with a platelet count of 316 × 10^9^/L. At the index presentation, his haemoglobin was 122 g/L, his white cell count was 7.37 × 10^9^/L with 30% lymphocytes, 56% neutrophils, and CRP was 24.4 mg/dL. He was clinically well but was admitted to the hospital with a working diagnosis of dengue fever due to the low platelet count.

His COVID-19 rapid antigen test was negative and RT-PCR did not detect SARS-CoV-2 when checked before admission. His Dengue NS1 antigen and Dengue IgM antibodies were negative, but his IgG antibodies were positive. His liver transaminases were normal. His US scan of the abdomen also did not show any evidence suggestive of dengue fever nor lymphadenopathy or organomegaly. The patient did not have a fever after the first day, nor was he clinically ill. But the platelet count persisted around 10 × 10^9^/L, with normal white and red cell counts, bilirubin, and blood picture.

On focused questioning and investigation to look for the cause for low platelets, cytomegalovirus and Epstein–Barr virus antibodies and hepatitis A, B, and C screenings were negative. Further questioning revealed that his father had been diagnosed with COVID-19 four weeks ago and this boy also had a positive COVID-19 real-time PCR test with a 27 Ct value, but he had not had any symptoms of COVID-19 at that time. Asymptomatic COVID-19 infection was reconfirmed by having a high anti-SARS-CoV-2 antibody level at 125.3 ng/mL (positive ≥0.8 ng/mL). His blood picture was reported as having a severe bacterial infection or autoimmune disease, but serum procalcitonin was 0.6 ng/ml. There were no abnormal cells in the blood picture. His lactate dehydrogenase (LDH) was marginally elevated at 441 U/L (normal 85–227), ANA was positive (1 : 160), but bilirubin and the direct Coomb test were negative. He did not have symptoms or signs suggestive of any other autoimmune disease. His rheumatoid factor level was normal and antidouble stranded DNA (anti-dsDNA) test was negative. His d-Dimer was high at 3000 ng/mL, but ferritin was normal at 150 micrograms/L, and there was no evidence of deep venous thrombosis in the venous Doppler scan of the legs. CT pulmonary angiogram did not show pulmonary emboli. His prothrombin time (PT) and activated partial thromboplastin time (aPTT) were normal.

With the above investigations, the suspicion of dengue fever was ruled out. A clinical diagnosis of ITP was made by exclusion and the patient was started on methylprednisolone 1 g daily for three days. His platelet count improved to 100 × 10^9^/L in 3 days, and the d-Dimer level dropped to normal (450 ng/mL) in 3 days following the initiation of methylprednisolone. Therefore, he was discharged home on prednisolone 40 mg daily (1 mg/kg, 42 kg).

**Figure 1:**
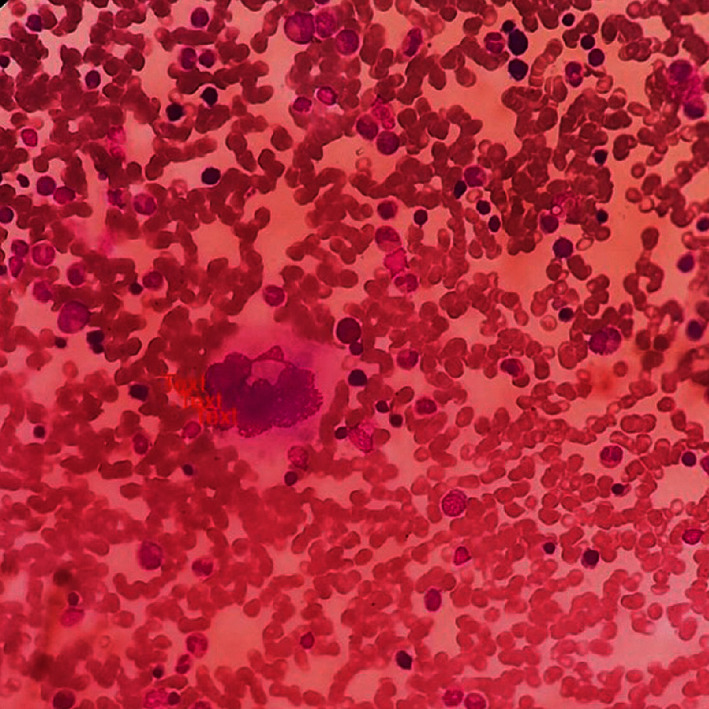
Bone marrow biopsy showing young increased megakaryocytes (haematoxylin and eosin-stained section, 100x magnification).

His platelet count after one week improved with prednisolone treatment but the best was 70 × 10^9^/L. But he was clinically well. A repeat blood picture done after 2 weeks showed steroid effects and did not point to any other diagnosis, but the platelet count dropped to 50 × 10^9^/L despite being on steroids. Therefore, his steroids were stopped, and a bone marrow biopsy was performed to exclude any other bone marrow pathology and to aid confirm the diagnosis of ITP. His platelet count dropped to 5 × 10^9^/L while off steroids awaiting a bone marrow biopsy but did not have any significant bleeding apart from a few mucosal bleeding spots in the mouth. His bone marrow biopsy did not show evidence of any haematological malignancy and was keeping with ITP, which is characterized by young, immature, less polyploid megakaryocytes (Figure 1).

The diagnosis of ITP secondary to asymptomatic COVID-19 was confirmed, and he was re-started on prednisolone. Due to his poor response to the second challenge with prednisolone, he was then started on azathioprine 50 mg bd. Following the initiation of azathioprine, his platelet counts improved to hover around 40–70 × 10^9^/L. In March 2022, the boy developed gingivitis and a buccal infection. He dropped his platelet count to 30 × 10^9^/L and developed neutropenia. He was treated with IV meropenem and IV immunoglobulin while continuing azathioprine. He made a good recovery after one week of treatment to have a platelet count of 120 × 10^9^/L and was discharged home on azathioprine. The patient got re-admitted with pancytopaenia in 6 months and azathioprine was stopped at this second presentation. His pancytopaenia improved in one week and he was discharged home without any immunosuppressants.

The boy did not have any more hospital admissions, and he is currently not on any immunosuppressants. He is being followed up on monthly with FBC. He maintains platelet counts around 40,000–70,000 × 10^9^/L with normal white and red blood cell counts and is symptom-free.

## 3. Discussion

This case reports a persistent, relapsing, and remitting course of Immune thrombocytopenic purpura (ITP) secondary to asymptomatic COVID-19 infection in a child, which is poorly responsive to standard immunosuppressants.

This boy presented with isolated thrombocytopenia accompanied by a positive ANA, compatible bone marrow biopsy, and minor mucocutaneous bleeding four weeks following a confirmed asymptomatic COVID-19 infection. He did not have organomegaly or any evidence of acute infection, nor did he have any evidence of other viral infections that could have caused low platelet counts at the presentation. His red and white cell lines were not affected. There was no evidence of other noninfective causes for low platelets like disseminated intravascular coagulation (DIC), thrombotic thrombocytopenic purpura/haemolytic uraemic syndrome (TTP/HUS), or heparin-induced thrombocytopenia (HIT), and therefore, a diagnosis of ITP was made.

He had a partial response to first- and second-line immunosuppressive medications and is currently having a relapsing and remitting course of ITP nearly two years after the initial illness which is not common for post-COVID-19 ITP and not common for ITP in children either.

Secondary ITP following COVID-19 has been reported in a systematic review of 45 patients, and only three (7%) of them were children, and 71% were more than 50 years of age [2]. In the clinical presentation, 75% of patients had moderate-to-severe COVID-19, but only 7% were asymptomatic. The majority, 78%, of cases of ITP was detected during the COVID-19 illness, but 20% were detected three weeks following the COVID-19 infection. Good initial response to a short course of glucocorticoids and intravenous immunoglobulin has been noted, and a delayed response has been reported in only one case which again was in an adult. Of them, 26 (58%) patients had a complete response with a platelet count of more than 100 × 10^9^/L. Patients responded to steroids or immunoglobulins and only four patients (9%) relapsed. The median number of days to the platelet response was five days [2]. Cleveland Clinic's experience report outcome and management of 11 patients with ITP secondary to COVID-19; mean age of 63 years, with a complete response in 45.5% and response to treatment in 27.3% of patients with steroids and immunoglobulin, with a median recovery time of four days [3]. Another review observed 57 patients with a mean age of 56 years, where they observed significantly lower platelet counts and higher bleeding rates but an excellent response to the first-line therapies in 76.9% compared to other ITP series [4].

There were no abnormal cells in the blood picture, nor was organomegaly or lymphadenopathy to account for a haematological malignancy except for marginally elevated LDH. There was no evidence of other viral infections that could cause thrombocytopenia The quick platelet response to intravenous methylprednisolone was in favour of the diagnosis of ITP, but the subsequent observation of partial response to steroids made us proceed with a bone marrow biopsy which excluded any other cause for low platelets confirming the diagnosis of ITP. However, the partial response to usual immunosuppressant treatment observed in this case emphasizes the need for more understanding of the mechanism of causation of low platelets following COVID-19.

The behaviour of platelets in COVID-19 is interesting [5, 6]. Mild to moderate disease may have normal or slightly high platelets. Platelet drop is always seen in most critically ill patients. Several theories have been postulated for the pathophysiology of low platelets in COVID-19: a reduction in platelet production, an increase in platelet destruction, or a decrease in circulating platelets [7]. Impaired platelet production is thought to be due to bone marrow suppression induced by the cytokine storm or direct infection of the hematopoietic cells. Increased platelet destruction is believed to be due to increased destruction caused by antibodies and immune complexes. A reduction in circulating platelets in the bloodstream is also postulated and is thought to be secondary to an intense lung injury causing a pulmonary intravascular coagulopathy bout our patient had none of those.

Mild thrombocytopaenia is a common finding with many viral infections. This is thought to be secondary to inflammation-induced platelet consumption, destruction, sequestration, defective production or myelo-suppression [8]. All these usually settle with the setting of inflammation. Acute ITP following viral infections is especially reported of children in literature and this too settles in about 6 months following the triggering infection. Platelets are increasingly recognized as having an active role in the immune response of the innate and adaptive immune system and also with viruses. The main mechanism of ITP is believed to be due to antibody-mediated and/or T cell-mediated platelet destruction. However, in addition, impairment of T cells, cytokine imbalances, and the contribution of the bone marrow niche have now been recognized as important contributors [9].

According to the American Society of Haematology (ASH) 2019 guidelines for immune thrombocytopenia [10], in children with newly diagnosed ITP, without life-threatening bleeding, the first-line treatment suggested is corticosteroids rather than immunoglobulins. Prednisolone is preferred over dexamethasone in a dose of 2–4 mg/kg per day; a maximum of 120 mg daily, for 5–7 days; and recommended against courses of corticosteroids longer than seven days. When first-line therapy fails, the panel suggests the use of a thrombopoietin receptor agonist (TPO-RA) rather than rituximab or splenectomy, which is not freely available in low-middle income countries. Hence, we have used azathioprine.

## 4. Conclusions

Persistent, relapsing, and remitting course of immune thrombocytopenic purpura (ITP) secondary to asymptomatic COVID-19 infection which is poorly responsive to standard immunosuppressants in children is rare but is observed. This case emphasizes the importance of COVID-19 testing in asymptomatic patients with low platelets or newly diagnosed ITP patients to differentiate between primary and secondary ITP as the management and the course of the illness may vary with the diagnosis. On the other hand, it may not be unwise to follow-up COVID-19 survivors with a platelet count at around four weeks from the index infection to identify people who develop secondary ITP without many symptoms. Further, the observed poor response to standard immunosuppression warrants more research into the understanding of the pathophysiology of persistent low platelets following COVID-19 infection.

## Data Availability

The data that support the findings of this study are available from the corresponding author upon reasonable request.
